# IL-6 and IL-27 play both distinct and redundant roles in regulating CD4 T-cell responses during chronic viral infection

**DOI:** 10.3389/fimmu.2023.1221562

**Published:** 2023-07-31

**Authors:** James A. Harker, Trever T. Greene, Burton E. Barnett, Phuc Bao, Aleksandr Dolgoter, Elina I. Zuniga

**Affiliations:** ^1^ Division of Molecular Biology, Department of Biological Sciences, University of California San Diego, La Jolla, CA, United States; ^2^ National Heart and Lung Institute, Imperial College London, London, United Kingdom

**Keywords:** chronic viral infection, lcmv, IL-6, IL-27, T follicular helper cells

## Abstract

The IL-6 cytokine family signals through the common signal transduction molecule gp130 combined with a cytokine-specific receptor. Gp130 signaling on CD4 T cells is vital in controlling chronic infection of mice with lymphocytic choriomeningitis virus clone 13 (LCMV Cl13), but the precise role of individual members of the IL-6 cytokine family is not fully understood. Transcriptional analysis highlighted the importance of gp130 signaling in promoting key processes in CD4 T cells after LCMV Cl13 infection, particularly genes associated with T follicular helper (Tfh) cell differentiation and IL-21 production. Further, *Il27r^−/−^Il6ra^−/−^* mice failed to generate antibody or CD8 T-cell immunity and to control LCMV Cl13. Transcriptomics and phenotypic analyses of *Il27r^−/−^Il6ra^−/−^* Tfh cells revealed that IL-6R and IL-27R signaling was required to activate key pathways within CD4 T cells. IL-6 and IL-27 signaling has distinct and overlapping roles, with IL-6 regulating Tfh differentiation, IL-27 regulating CD4 T cell survival, and both redundantly promoting IL-21.

## 1 Introduction

Chronic viral infections, such as HIV-1 and hepatitis C virus (HCV) in humans or lymphocytic choriomeningitis virus (LCMV) in mice, pose a distinct challenge to the host. Their persistent replication results in recalibration of the host’s immune response, as it seeks to restrain viral replication while limiting immune-associated pathology to the host (1). This is often characterized by deletion and functional exhaustion of virus-specific CD8 and CD4 T cells, including suppression of IFN-γ, TNF, and IL-2 production (1, 2). In contrast, T follicular helper (Tfh) cells, a specialized subset of CD4 T cells that support germinal center (GC) B cells, expand during the later stages of chronic infection (3–6), enhancing virus-specific antibody responses and promoting viral containment (7, 8).

Cytokine signaling plays a vital role in this re-calibration process; regulatory cytokines such as IL-10 and TGF-β have long been known to limit virus-specific CD8 T-cell and CD4 T-cell numbers and enable their functional exhaustion [reviewed in (1, 2)]. Type I interferon (IFN-I) signaling, while normally antiviral, when sustained at later stages of infection, has been shown to also favor viral persistence by limiting T-cell responses (9, 10). Conversely, IL-6, IL-21, and IL-27 are required for viral control during chronic infection (11–15). In particular, IL-21 is a critical regulator of antibody-mediated immunity in response to infection or vaccination (16), specifically through the action of Tfh-derived IL-21 on GC B cells, which promotes their function and survival (17). In addition, during persistent infection with LCMV clone 13 (Cl13), IL-21 receptor signaling on CD8 T cells is also critical for virus-specific CD8 T-cell survival and function, as well as viral control (11–13). This process appears to be dependent on IL-21-STAT3-mediated upregulation of BATF and IRF4-mediated BLIMP1 expression in virus-specific CD8 T cells (18, 19). Notably, IL-21 production by CD4 and CD8 T cells is also associated with enhanced CD8 T-cell responses and viral control in individuals with HIV-1 and HCV infections (20–22).

IL-6 and IL-27, both members of the IL-6 family of cytokines, influence CD4 T-cell responses during chronic infection but in distinct fashions. IL-6 deficiency results in reduced Tfh differentiation and antibody-mediated immunity (14), a process in part dependent on IL-6 signaling on CD4 T cells to sustain Tfh formation and function at the later stages of infection (14, 23). However, IL-27 is required to maintain virus-specific CD4 T-cell numbers after chronic viral infection (15) but also influences IFN-I, DC, and NK cell responses early after infection (24). Signaling on CD4 T cells *via* the common signal transduction molecule gp130 (*Il6st*), which all members of the IL-6 family of cytokine used to mediate signaling, appears to be critical for the effects of both IL-6 and IL-27 in chronic viral infection (15). Intriguingly, while mice deficient in either IL-6 or IL-27 signaling show no reduction in IL-21 expression, signaling through gp130 is critical for IL-21 production by CD4 T cells in chronic viral infection. Indeed, gp130 deficiency in T cells results in significantly reduced IL-21 expression in CD4 T cells as well as limited virus-specific CD8 T-cell numbers and function, phenocopying IL-21 deficiency (15, 23). Given the importance of sustained antiviral immunity in controlling persistent viral infections, here, we sought to dissect the precise roles of IL-6 and IL-27 in the regulation of gp130-mediated responses.

## 2 Materials and methods

### 2.1 Mice and viral stocks

Wild-type (WT) C57BL/6, WT CD45.1^+^ (B6.SJL-*Ptprc^a^ Pepc^b^*/BoyJ), and *Il27ra^−/−^* (WSX-1, alternatively named IL-27R deficient) mice were purchased from The Jackson Laboratory (Bar Harbor, ME, USA). *Il6ra^−/−^* (IL-6Ra-deficient) mice on a C57BL/6 background were kindly provided by Dr. Angela Drew (University of Cincinnati, USA). *Cd4-*cre *Il6st^fl/fl^* (T cell-specific gp130-deficient) mice were originally provided by Dr. Werner Mueller (University of Manchester, UK). *Il6ra^−/−^* mice were crossed with *Il27ra^−/−^* mice to generate *Il6ra^−/−^ Il27ra^−/−^* (double deficient; hereafter referred to as dKO) mice. All mice were bred and maintained in a closed breeding facility, and mouse handling conformed to the National Institutes of Health and the Institutional Animal Care and Use Guidelines of UCSD. For the studies using whole dKO mice, 6–8-week-old mice were infected intravenously (i.v.) with 2 × 10^6^ pfu of LCMV Cl13. For mixed bone marrow (BM) chimeras, recipient 6–8-week-old CD45.1^+^ mice were exposed to 1,000 rads. The following day, they were injected with 5 × 10^6^ BM cells i.v. (a mix of 50% CD45.1^+^ WT and 50% of CD45.2^+^ WT, *Cd4*-cre *Il6st^fl/fl^*, *Il27ra^−/−^*, *Il6ra^−/−^*, or *Il6ra^−/−^Il27ra^−/−^* donor cells). Mice were maintained on oral antibiotics for 2 weeks, and reconstitution was continued for an additional 6 weeks prior to infection. LCMV Cl13 was grown, identified, and quantified as previously described (14). Viral quantification was carried out using a six-well plate plaque assay on VERO cells (ATCC).

### 2.2 Flow cytometry

Spleens were incubated with 1 mg/ml of collagenase D (Roche, Basel, Switzerland) at 37°C for 20 min and passed through a 100-μm cell strainer before being counted on a Coulter counter. Flow cytometry was then carried out as previously described. Anti-mouse antibodies were obtained from BioLegend (San Diego, CA, USA) unless otherwise stated: anti-CD8-Pacific blue, anti-CD4-allophycocyanin-Cy7, anti-CD19-PE, anti-B220-PE-CF594, anti-CD38anti-Alexa Fluor 700, anti-CD38-PE-Cy7, anti-GL7-FITC, anti-GL7-efluor660, anti-CD138-PE, anti-IgM-allophycocyanin-Cy7, anti-IgD-PB, anti-PD1-PE-Cy7, anti-PD1-BV605, anti-ICOS-PE, anti-ICOS-PE-Cy7, anti-CD11a-FITC, anti-CD49d-PerCP-Cy5.5, anti-KLRG1-FITC, anti-CD127-PerCP-Cy5.5, anti-CD8-efluor450, anti-CD45.1-PE-CF594, anti-CD45.2-BV650, anti-IgG1-FITC, and anti-IgG2a^b^-biotin followed by Strepavidin-BV650, anti-IFN-γ-allophycocyanin, anti-TNF-α-FITC, anti-IL-2-PE, and anti-CXCR5-BV421. Foxp3 (eBioscience, San Diego, CA, USA) staining and Bcl6 (K112-91; BD Biosciences, San Jose, CA, USA) staining were carried out after fixation with intranuclear fixation/permeabilization buffer (eBioscience). IL-21 staining was carried out by two-step intracellular staining, first, with 1:50 dilution of recombinant mouse IL-21R human Fc chimera protein (R&D Systems, Minneapolis, MN, USA), followed by 1:200 anti-human Fc-PE (BioLegend). Biotinylated D^b^ GP_33–41_ and D^b^ NP_396–404_ monomers along with allophycocyanin-I-A^b^ GP_67–77_ tetramers were kindly provided by the National Institutes of Health Tetramer Core Facility (Atlanta, GA, USA). Monomers were folded using SA-PE or SA-allophycocyanin (Molecular Probes, Life Technologies, Carlsbad, CA, USA).

### 2.3 Fluorescence-activated cell sorting

For analysis of antigen-specific splenocytes, cells were prepared for flow cytometry. Splenocytes were then enriched for CD4 T cells using a negative CD4 T cells isolation kit (STEMCELL Technologies, Vancouver, BC, Canada). For CD45.1^+^:*Cd4*cre*Il6st^fl/fl^* mice, cells were then stained with antibodies specific for CD45.1, CD45.2, CD4, and PD1 for 30 min at 4°C, and CD45.1^+^CD4^+^PD1^+^ (WT) and CD45.2^+^CD4^+^PD1^+^ (gp130-deficient) cells were fluorescence-activated cell sorting (FACS) isolated. For CD45.1^+^:*Il6ra^−/−^Il27ra^−/−^* mice, cells were then stained with antibodies specific for CD45.1, CD45.2, CD4, PD1, SLAM, CD62L, and CXCR5, and CD45.1^+^CD4^+^PD1^+^CXCR5^+^SLAM^−^ (WT) and CD45.2^+^CD4^+^PD1^+^ CXCR5^+^SLAM^−^ (dKO) cells were FACS isolated.

### 2.4 LCMV-specific antibody ELISAs

LCMV-specific enzyme-linked immunosorbent assays (ELISAs) were performed as we and others have previously described using antigen prepared by purifying LCMV on a Renografin gradient (14). Endpoint titers were calculated by determining the lowest dilution at which a specific antibody was at least two times the standard deviation (SD) above the background.

### 2.5 Microarray analysis

RNA was isolated using RNeasy kits (Qiagen, Valencia, CA, USA) and hybridized on a Mouse Gene 1.0 ST Array platform (Affymetrix, Santa Clara, CA, USA). Bioconductor Oligo package was used for pre-processing data (RMA normalization) (25). Expression sets were then filtered using the R package genefilter (26) to exclude values with low expression or variance, as well as probes that did not correspond to an ENTREZ entry. Data were then subjected to paired analysis with Limma (Linear Models for Microarray Analysis) to determine differential gene expression (27, 28). A false discovery rate cutoff of 0.1 (T-Δgp130) or 0.25 (dKO) was used to define differentially expressed genes between the conditions. Overrepresentation analysis (ORA) was performed with the WebGestalt platform using a false discovery rate (FDR) significance level of 0.05 (29) and a background data set containing all of the genes for which expression was evaluated on our microarray platform. The transcriptomic data have been deposited in the GEO repository (GSE235035).

### 2.6 Real-time PCR

Total RNA was extracted from splenocytes using RNeasy kits (Qiagen) and reverse transcribed into cDNA using superscript III RT (Invitrogen, Carlsbad, CA, USA). cDNA quantification was performed using SYBR Green PCR kits (Applied Biosystems, Foster City, CA, USA) and a Real-Time PCR Detection System (Applied Biosystems Innovation). The primers used were as follows:

**Table:** 

Gene name	Forward sequence	Reverse sequence
*Gapdh*	catcactgccacccagaagactg	atgccagtgagcttcccgttcag
*Il6ra*	tgcagttccagcttcgataccg	tgcttcactcctcgcaaggcat
*Il27ra*	ccaacctgtctctggtgtgctt	tactccaacggtttcctggtcc
*Il21*	gcctcctgattagacttcgtcac	caggcaaaagctgcatgctcac
*Nlrc5*	cttcccgcctctccttccacaat	ctccacctgcccacatcctacca
*Tcf7*	cctgcggatatagacagcacttc	tgtccaggtacaccagatccca
*Ube2a*	acagtccactttgtgcgaga	ttagggcaacactgctcctt
*Ube2v1*	aactagctctccggtaggca	agtagactcggacgacgaca

### 2.7 Confocal microscopy

Spleen segments were immediately fixed with 4% paraformaldehyde and 5% sucrose, followed by 10% and then 30% sucrose overnight. The segments were then flash-frozen in an OCT compound (Tissue Tek). Sections measuring 7 μm were cut, refixed with 4% paraformaldehyde, and then stained with Abs (IgD, clone 11-26C; GL7, clone GL7; CD4, clone RM4-5; B220, clone RA3-6B2). Enumeration of GCs was performed by scoring the presence of a cluster of GL7^+^ B cells within a B220^+^ B-cell zone. Three B-cell zones were scored per mouse, and the average number of GCs per B-cell zone was calculated. Confocal images were captured with an Olympus FV1000. Individual GC images were captured at ×40.

### 2.8 Statistics

Statistical analysis was carried out using GraphPad Prism 5.0 (GraphPad, La Jolla, CA, USA). For comparisons between the two groups, a non-parametric Mann–Whitney U test was performed. For pairwise analysis, a Wilcoxon test was used.

## 3 Results and discussion

### 3.1 Gp130 signaling on CD4 T cells drives a follicular helper cell signature during chronic viral infection

**Figure 1:**
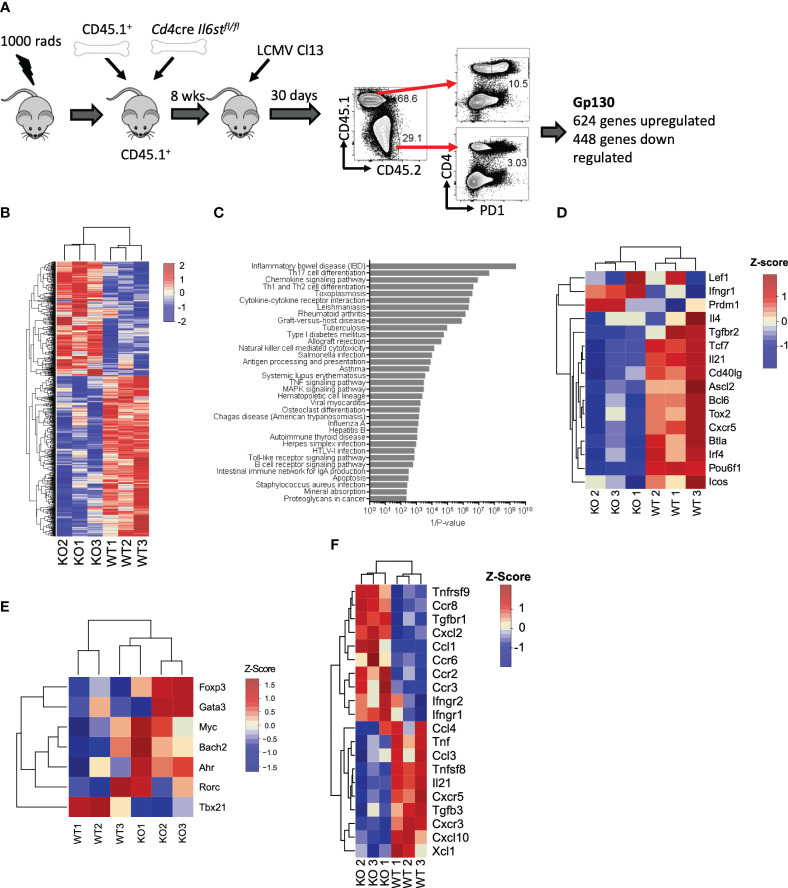
T cell-intrinsic gp130 signaling resulted in a Tfh differentiation signature in chronic viral infection. Mixed CD45.1^+^ (WT): CD45.2^+^ *Cd4*cre *Il6st^fl/fl^* (T-Δgp130) bone marrow chimeras were infected i.v. with 2 × 10^6^ pfu of LCMV CL13, and 30 days later, CD45.1^+^ and CD45.2^+^ PD1^+^ CD4^+^ T cells were FACS isolated and transcriptomics carried out. **(A)** Schematic of experiment. **(B)** Heatmaps of significantly differentially expressed genes between CD45.1^+^ and CD45.2^+^ compartments. **(C)** ORA of KEGG pathways. **(D)** Heatmaps showing genes differentially expressed in WT versus KO cells associated with KEGG pathways. **(E)** Heatmaps of selected genes known to be associated with Tfh and other CD4 T helper subsets. **(F)** Heatmap of differentially expressed chemokine and cytokines and their receptors. Data represent three independent experiments of at least five mice per group. LCMV CL13, lymphocytic choriomeningitis virus clone 13; FACS, fluorescence-activated cell sorting; ORA, overrepresentation analysis; KEGG, Kyoto Encyclopedia of Genes and Genomes; WT, wild type; KO, knockout.

We first unbiasedly evaluated the overall role of the IL-6 cytokine family on CD4 T cells during LCMV Cl13 infection. For that, we generated mixed BM chimeras with CD45.2^+^ *Cd4*-cre *Il6st* ^fl/fl^ (T cell-specific gp130 deficient) and CD45.1^+^ WT mice and performed transcriptomic analysis of gp130-deficient or WT antigen-experienced splenic PD1^+^ CD4 T cells at day 30 post-infection (p.i.) (**Figure 1A**). We noted that although irradiated recipients were reconstituted with equal numbers of WT and *Cd4*cre*Il6st^fl/fl^* BM, 8 weeks after generation, and even before infection, CD45.1^+^ peripheral blood mononuclear cells (PBMCs) were more prevalent than CD45.2^+^ PBMCs [**Figure 1A** and (15)]. Gp130 deletion resulted in over 1,000 differentially expressed (DE) genes, 624 genes upregulated and 448 genes downregulated (**Figure 1B** and **Supplementary Table 1**). ORA of Gene Ontology (GO) revealed that several core immunological processes were dysregulated in the absence of gp130, with T helper cell differentiation and cytokine signaling being among the most differentially regulated (**Figure 1C**). Fitting with altered T helper cell differentiation and previous findings that loss of gp130 significantly reduced the number of Tfh generated after LCMV Cl13 infection (15), genes known to be associated with Tfh differentiation and function, including *Bcl6*, *Tcf7*, *Tox2*, *Irf4*, *Cxcr5*, *Il21*, and *Icos*, were among the most downregulated in CD4 T cells lacking gp130 (**Figure 1D**). Although not significant, gp130 deficiency led to a trend for increased expression of the lineage-defining transcription factors for Th2 (*Gata3*), Th17 (*Rorc*, *Ahr*, and *Myc*), and Tregs (*Foxp3*) and reduced Th1 (*Tbx21*) (**Figure 1E**). Other cytokine and chemokine receptors, outside the IL-6 family, were also modulated, with *Ifngr1*, *Ifngr2*, and *Tgfbr1* upregulated in the absence of gp130, while *Tfgbr3*, as well as *Cxcr3* and its ligand *Cxcl10*, was downregulated (**Figure 1F**). Taken together, these data suggest that gp130 signaling on CD4 T cells shapes their differentiation and how they respond to the extracellular milieu, in particular promoting Tfh responses, during chronic viral infection.

### 3.2 IL-6Ra and IL-27Ra signaling are essential for virus-specific T-cell and antibody responses after LCMV Cl13 infection

**Figure 2:**
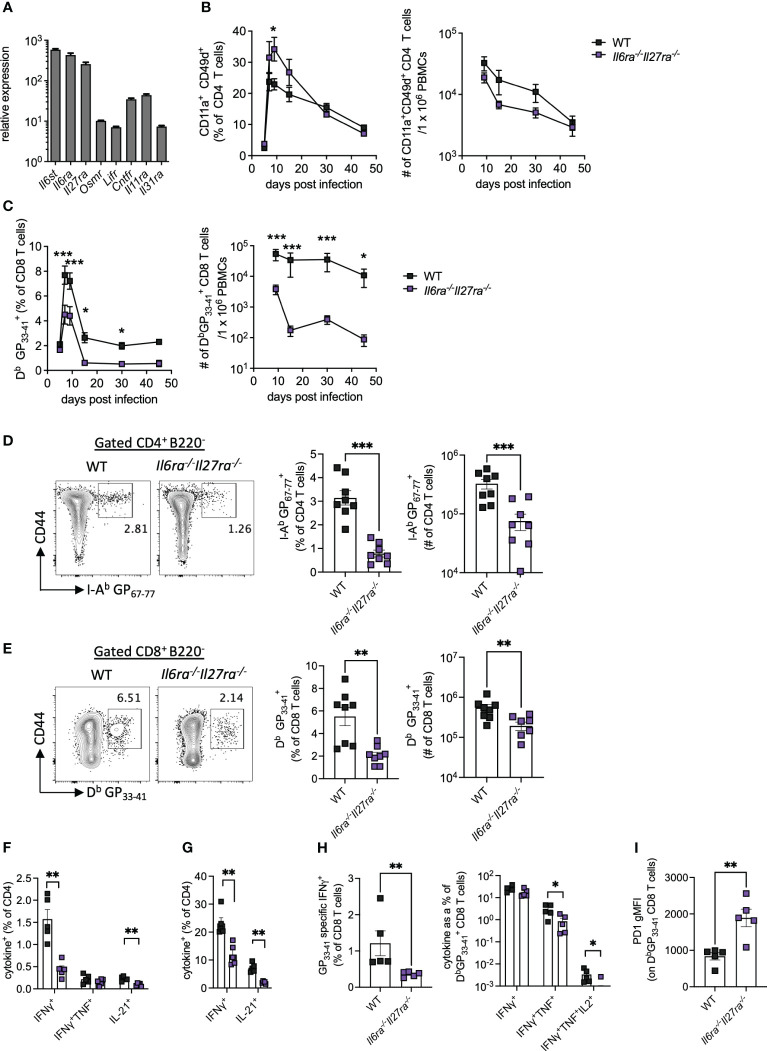
IL-6R and IL-27R were required for optimal virus-specific T-cell responses during chronic viral infection. **(A)** Expression of IL-6 cytokine family receptor transcripts in splenic WT PD1^+^ CD4^+^ T cells at day 30 post-LCMV Cl13 infection measured by microarray as described in **Figure 1**. **(B–I)** WT (C57BL/6) and *Il6ra^−/−^Il27ra^−/−^* (dKO) were infected with 2 × 10^6^ pfu of LCMV Cl13 i.v. **(B)** CD11a^+^CD49d^+^ CD4 T cells and **(C)** D^b^GP_33-41_-specific CD8 T cells in the blood at multiple timepoints post-infection. **(D–I)** At day 30 p.i., the following parameters were assessed in spleen *via* flow cytometry: I-A^b^ GP_67-77_ CD4 T cells, **(D)** D^b^GP_33-41_ CD8 T cells **(E)**, CD4 T-cell cytokine production after GP_61-80_ peptide stimulation **(F)** and PMA/I stimulation **(G)**, CD8 T-cell IFN-γ production and functional exhaustion (% cytokine^+^/D^b^GP_33-41_^+^) after GP_33-41_ peptide stimulation **(H)** and PD1 expression on D^b^GP_33-41_ CD8 T cells **(I)**. Data are representative of n > 5 mice per timepoint and two independent repeats. **(B–I)** Mann–Whitney U test was used; *p < 0.05, **p < 0.01, and ***p < 0.001. WT, wild type; LCMV CL13, lymphocytic choriomeningitis virus clone 13; PMA/I, phorbol myristate acetate and ionomycin.

Analysis of the transcriptomic data revealed that *Il6st*, *Il6ra*, and *Il27ra* were the most abundantly expressed receptors of the IL-6 cytokine family in WT (CD45.1^+^) PD1^+^ CD4 T cells at day 30 p.i., as indicated by at least a 10-fold increased expression than other receptors analyzed (**Figure 2A**). Global genetic ablation of *Il6*, *Il27ra*, or *Il6st* in mice each has distinct outcomes on the ensuing immune response upon LCMV Cl13 infection, and most intriguingly, individual deletion of *Il6ra* and *Il27ra* did not fully recapitulate the cell-intrinsic effects of gp130 deletion in CD4 T cells (e.g., defective IL-21 production observed in gp130 ko CD4 T cells) (14, 23, 24). One possible explanation for this observation is the existence of redundant effects between IL-6 and IL-27 signaling in CD4 T cells. To investigate the combined effects of IL-6 and IL-27 deficiencies, we generated *Il6ra^−/−^Il27ra^−/−^* double knockout (dKO) mice and studied their immune responses upon LCMV Cl13 infection.

The proportion of antigen-experienced CD11a^+^ CD49d^+^ activated CD4 T cells, which reflect virus-specific CD4 T-cell responses during LCMV infection (30), was elevated in the blood of dKO mice compared to controls at early timepoints p.i., but similar proportions were seen from day 30 p.i. onward (**Figure 2B**). Importantly, the total number of antigen-experienced CD4 T cells in the blood of dKO mice was, however, similar to or slightly lower than that seen in WT mice (**Figure 2B**). However, both percentages and numbers of circulating LCMV-specific D^b^ GP_33-41_>^+^ CD8 T-cell responses were significantly reduced in dKO mice compared to WT controls from day 7 onward (**Figure 2C**), a phenotype that was not observed in individual IL-16- or IL-27-deficient mice (14, 24). Meanwhile, in the splenic compartment, virus-specific CD4 and CD8 T cells were significantly reduced in dKO mice compared to WT mice at day 30 p.i. (**Figures 2D, E**).

As with many other chronic viral infections, LCMV Cl13 infection results in progressive T-cell exhaustion and phenotypic alteration (31). This is characterized by the deletion of virus-specific CD8 T cells, alongside their functional impairment of those that remain, including the hierarchical loss of cytokine-secreting capacity in response to stimulation (32). CD4 T cells meanwhile switch from a predominantly Th1-like toward a Tfh-like IL-21-producing phenotype (1, 3, 11).

In the absence of IL-6 and IL-27 signaling, IFN-γ and IL-21, but not TNFα, from both virus-specific and polyclonally activated CD4 T cells were decreased when compared to WT mice, suggesting functional impairment of both Th1 and Tfh-like responses in dKO versus WT mice (**Figures 2F, G**). Likewise, virus-specific CD8 T cells from dKO mice also displayed a more exhausted phenotype with reduced hierarchical production of TNFα and IL-2 but not IFN-γ, when cytokine production after GP_33-41_ stimulation, normalized to total D^b^GP_33-41_^+^ CD8 T-cell numbers in the same animal, was compared to their WT counterparts (**Figure 2H**). Increased CD8 T-cell exhaustion is also associated with upregulation of the co-inhibitory molecule PD-1 (33, 34), the expression of which was enhanced on dKO *vs.* WT virus-specific CD8 T cells (**Figure 2I**).

**Figure 3:**
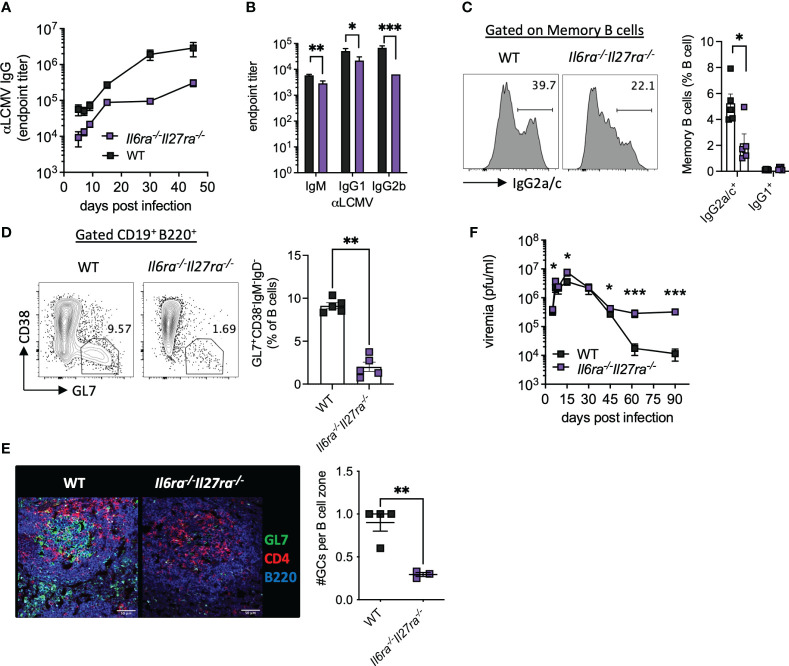
B-cell responses were compromised in the absence of IL-6 and IL-27 signaling. WT (C57BL/6) and *Il6ra^−/−^Il27ra^−/−^* were infected with 2 × 10^6^ pfu of LCMV Cl13 i.v. **(A)** Endpoint titers for serum anti-LCMV IgG quantified by ELISA. **(B)** Endpoint titers for anti-LCMV IgM, IgG1, and IgG2b at day 30 p.i. **(C, D)** Expression of IgG2a/c in gated memory B cells **(C)** and germinal center B cells **(D)** in the spleen at day 30 p.i. were assessed by flow cytometry. **(E)** Representative images of splenic CD4, GL7, and B220 expression at day 30 p.i. by confocal microscopy. A 50-μm scale bar is depicted. The number of germinal centers (GL7^+^) regions per B-cell zone imaged is enumerated. **(F)** LCMV viral load in the serum quantified by plaque assay. **(A–D, F)** Data are representative of n > 5 mice per timepoint and two independent repeats. **(E)** Data are representative of n = 3–4 mice from two independent repeats. Three B-cell zones were imaged per mouse, and the average GC/B-cell zone is shown. **(B–F)** Mann–Whitney U test was used; *p < 0.05, **p < 0.01, and ***p < 0.001. WT, wild type; LCMV CL13, lymphocytic choriomeningitis virus clone 13; ELISA, enzyme-linked immunosorbent assay.

Virus-specific antibody responses were also significantly reduced throughout infection in dKO mice compared to WT mice (**Figure 3A**). Analysis of antibody subtypes at day 30 p.i. revealed reduced IgM, IgG1, and in particular IgG2b in dKO mice compared to WT mice (**Figure 3B**). Consistently, IgG2a/c^+^ memory B cells and splenic GC were also significantly reduced in dKO mice at day 30 p.i. (**Figures 3C, D**), with GCs being largely decreased in the spleens of *Il6ra^−/−^Il27ra^−/−^* mice (**Figures 3D, E**).

In line with the aforementioned compromised B-cell and T-cell responses, loss of IL-6 and IL-27 signaling significantly increased viremia at days 5 and 15 p.i. and prevented viral control by days 60 and 90 p.i. (**Figure 3F**). Together, these data highlight the key roles that IL-6 and IL-27 signaling play in shaping antiviral immunity and promoting viral control during chronic viral infection.

### 3.3 CD4 T cell-intrinsic IL-6 and IL-27 signaling regulates the expression of multiple genes associated with Tfh function

**Figure 4:**
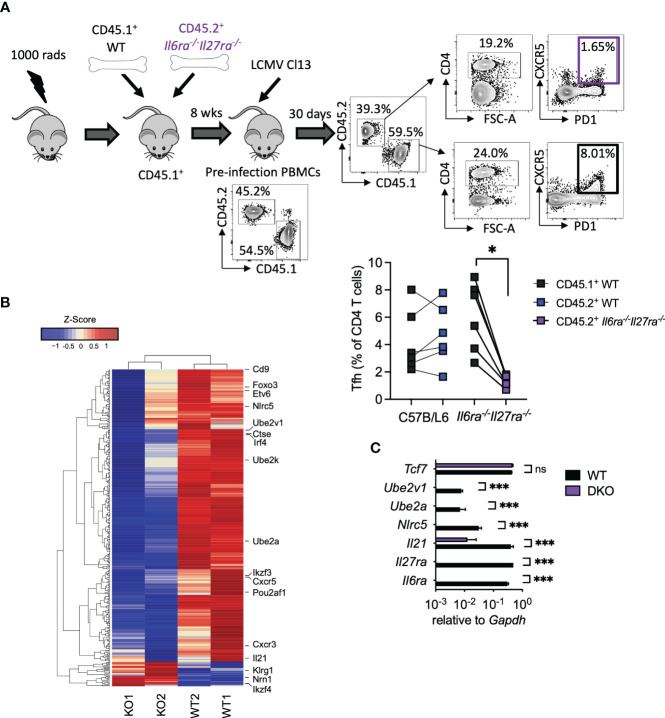
Cell-intrinsic IL-27 and IL-6 regulated transcriptional program in virus-specific Tfh during chronic infection. WT (C57BL/6) and *Il6ra^−/−^Il27ra^−/−^* were infected with 2 × 10^6^ pfu of LCMV Cl13 i.v., and CD45.1^+^ WT or CD45.2^+^ *Il6ra^−/−^Il27ra^−/−^* Tfh (CD4^+^PD1^+^SLAM^−^CXCR5^+^) was isolated by FACS from spleens of mixed BM chimeras at day 30 p.i. **(A)** A schematic plus representative flow cytometry plots are depicted including reconstitution of PBMCs prior to infection and spleen following infection. Graph depicts Tfh quantification within each BM compartment, and each symbol represents an individual mouse. **(B)** Transcriptomics analysis of T_FH_ was carried out. A heatmap of differentially regulated genes is shown. **(C)** RT-qPCR analysis of WT or *Il6ra^−/−^Il27ra^−/−^* Tfh isolated as in panel **(A)** Data represent n = 2 independent repeats, n > 4 mice per group. **(A)** Wilcoxon matched-pair signed-rank test; *p < 0.05. **(C)** Mann–Whitney U test was used; *p < 0.05, and ***p < 0.001. WT, wild type; ns, non-significant; LCMV CL13, lymphocytic choriomeningitis virus clone 13; FACS, fluorescence-activated cell sorting; BM, bone marrow; PBMCs, peripheral blood mononuclear cells.

As combined IL-6R and IL-27R deficiency appeared to substantially impair humoral immune responses, in particular GC formation (**Figures 3D, E**), we next sought to understand the cell-intrinsic effects of IL-6/IL-27 signaling specifically within Tfh cells themselves. For that, mixed BM chimeras using a 1:1 ratio of WT and dKO cells were generated (**Figure 4A**). As described above for WT: *Cd4*cre*Il6st^fl/fl^* mixed BM chimeras -**Figure 1A** and (15)], 8 weeks after generation, CD45.1^+^ PBMCs were slightly more prevalent than CD45.2^+^ PBMCs, with a consistent imbalance observed in the spleen at day 30 p.i. (**Supplementary Figure 1**). At day 30 p.i., Tfh (CD4^+^PD1^+^SLAM^−^CXCR5^+^) cells were readily detectable in both the WT and dKO CD4 T cell compartments but were significantly less frequent in the absence of IL-6Ra and IL-27Ra signaling (**Figure 4A**). To investigate potential Tfh qualitative differences (beyond the aforementioned numerical reduction) driven by a lack of IL-6 and IL-27 signaling, we performed a transcriptional analysis of FACS-purified Tfh cells isolated from the WT and dKO compartments. We observed that simultaneous loss of both IL-6 and IL-27 signaling resulted in downregulated expression of 332 genes and upregulated expression of 28 genes in dKO versus WT Tfh cells. While the expression of core transcription factors known to be involved in Tfh differentiation such as *Bcl6*, *Tcf7*, *Tox2*, and *Ascl2* was not significantly altered (in line with both CXCR5^+^PD1^+^SLAM^−^ populations sorted being Tfh), other genes involved in Tfh function were downregulated in the absence of both cytokines (**Figure 4B** and **Supplementary Table 2**). Most notably, *Il21* expression was one of the most downregulated genes in dKO Tfh cells, as were the transcription factors *Irf4* (IRF4), *Ikzf3* (Aiolos), *Foxo3* (Foxo3), and *Pou2af1* (Bob.1), which are known to routinely be upregulated in Tfh compared to other CD4 T cell subsets (35), and genetic deletion of each of these genes leads to reduced Tfh differentiation *in vivo* (18, 36–39). Within activated cells, both IRF4 and Aiolos have been found to interact with STAT3, while Foxo3 can bind the *Il21* promoter and, thus, are capable of regulating both production of and response to IL-21 (18, 38–40). *Cxcr5*, *cxcr3*, *Cd9*, and *Etv6*, other genes that are upregulated in Tfh cells (35), also appear to be cell-intrinsically promoted by IL-6 and IL-27 signaling. Interestingly, *Cd9* and *Cxcr3*, along with *Il21*, *Irf4*, and *Il6ra* itself, are all regulated by UTX, a histone 3 lysine 27 demethylase that is essential for the Tfh-dependent clearance of LCMV Cl13 (41). UTX appears to operate by upregulating Tfh responsiveness to IL-6 at the latter stage of infection, which is required for viral clearance (14). Among other genes that were downregulated in the absence of IL-6Ra and IL-27ra signaling were the ubiquitin-conjugating enzyme E2 genes (*Ube2a* and *Ube2v1*), *Nlrc5*, and *Ctse*.

Upregulation of gene expression in the absence of IL-6Ra and IL-27Ra was much less frequent; however, three notably upregulated genes were *Ikzf4* (Eos), *Nrn1* (Neuritin-1), and *Klrg1* (KLRG1). Eos acts in an antagonist fashion to Aiolos (38, 42) and is thereby consistent with the aforementioned downregulation of Aiolos in dKO Tfh cells. Neuritin-1 production by follicular regulatory T cells (Tfr) has recently been shown to be a negative regulator of IgE switch and also the differentiation of GC B cells into antibody-secreting plasma cells (43). Meanwhile, the inhibitory receptor KLRG1 is usually associated with lymphocytes, including CD4 T cells, acquiring a cytotoxic-like program, and we found that is negatively regulated by IL-27 and TGF-β signaling in chronic infection (44, 45). RT-qPCR analysis of selected genes in WT and dKO Tfh validated these results, with *Il6ra* and *Il27ra* being undetectable in dKO Tfh (**Figure 4C**). Likewise, *Il21*, *Nlrc5*, *Ube2a*, and *Ube2v1* were significantly downregulated or undetectable in dKO versus WT Tfh cells, while *Tcf7* expression was unaffected (**Figure 4C**). Together, these data supported the conclusion that signaling through IL-6 and IL-27 is required for the expression of key genes involved in Tfh function, including IL-21, during chronic viral infection.

### 3.4 CD4 T cell-intrinsic IL-6 and IL-27 signaling regulates Tfh differentiation and IL-21 production

**Figure 5:**
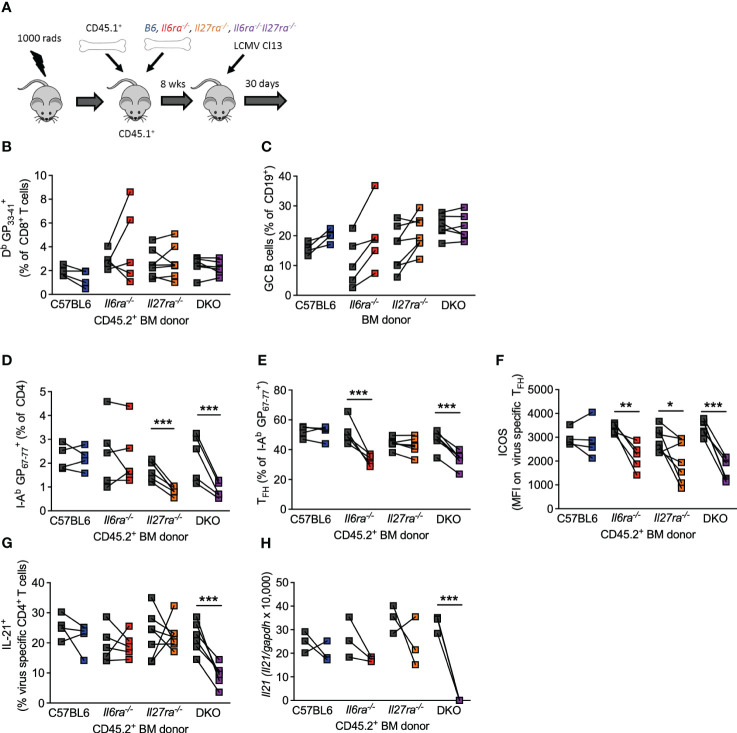
Intrinsic IL-6 and IL-27 signaling on CD4 T cells regulated overlapping and distinct functions. Mixed BM chimeras (using CD45.2^+^ C57/B6, *Il6ra* ^−/−^, *Il27ra^−/−^*, or *Il6ra^−/−^Il27ra^−/−^* donors) were infected with 2 × 10^6^ pfu of LCMV Cl13. **(A)** Schematic of the experiment is depicted. **(B–G)** At day 30 p.i., the frequency of virus-specific CD8 T cells **(B)**, germinal center B cells **(C)**, frequency of virus-specific CD4 T cells **(D)**, the proportion of T_FH_ (CXCR5^+^Bcl6^+^) **(E)**, the expression of ICOS on virus-specific T_FH_, **(F)** and proportion of IL-21^+^ CD4 T cells after GP_67-77_ peptide stimulation normalized to the frequency of I-A^b^ GP_67-77_^+^ CD4 T cells **(G)** were determined by flow cytometry. **(H)** CXCR5^+^PD-1^+^ CD4 T cells were FACS isolated, and *Il21* transcript was determined by RT-qPCR. **(A–E)** Three independent experiments from sorted pooled PD1^+^ CD4 T cells. **(H)** Three independent experiments on pooled sorted cells. **(B–H)** A Wilcoxon matched-pair test was used; *p < 0.05, **p < 0.01, and ***p < 0.001. BM, bone marrow; LCMV CL13, lymphocytic choriomeningitis virus clone 13; FACS, fluorescence-activated cell sorting.

The above data clearly demonstrate that IL-6 and IL-27 are critical for a range of Tfh cell functions after chronic viral infection and that combined ablation of both cytokine signaling had more profound effects on antiviral immunity than what was reported for ablation of each cytokine signaling on its own (14, 15, 24, 46). Thus, to compare side by side the individual versus combined (cell-intrinsic) effects of IL-6 and IL-27 receptor signaling in immune responses, we generated *Il6ra^−/−^*, *Il27ra^−/−^*, and dKO mixed BM chimeras and analyzed their B-cell and T-cell responses in parallel at day 30 p.i. (**Figure 5A**). As we have shown before (14, 15), the generation of virus-specific CD8 T cells and GC B cells was unaffected by the individual loss of IL-6Ra or IL-27Ra signaling (**Figures 5B, C**). Importantly, the combined loss of both cytokine signaling did not alter the number of antiviral CD8 T cells of GC B cells (**Figures 5B, C**), indicating that the aforementioned defects observed in these cell populations from dKO-infected mice were cell extrinsic. In addition, in line with previous studies (14, 15), intrinsic IL-27 signaling was required for the accumulation of virus-specific CD4 T cells (**Figure 5D**), and intrinsic IL-6 signaling was critical for the differentiation of virus-specific CD4 T cells into Tfh cells (**Figure 5E**). Meanwhile, signaling by both cytokines was needed for optimal ICOS expression by Tfh cells (**Figure 5F**). As expected, combined deficiency of both IL-6 and IL-27 signaling on CD4 T cells phenocopied each of these effects, with the reduced polyclonal Tfh frequencies observed in dKO mice (**Figure 4A**) also being seen in I-A^b^GP_67-77_^+^ CD4 T cells (**Figures 5D–F**). Importantly, neither IL-6 nor IL-27 was required for IL-21 production or *Il21* expression by virus-specific Tfh cells (**Figures 5G, H**), but the loss of both cytokine signaling resulted in a significantly reduced production of IL-21 after stimulation and undetectable *Il21* expression.

Overall, the results presented here highlight the critical roles that IL-6 and IL-27 play in shaping CD4 T-cell responses, especially Tfh, during chronic viral infection. Tfh can also be important in promoting protective immunity following acute viral infections (47), and likewise, IL-6, IL-27, and gp130 signaling has been shown to influence their formation and function, especially when acting in concert with IL-21 (23, 48, 49). Perhaps most importantly, we showed that IL-6 and IL-27 signaling acts redundantly to promote IL-21 production, answering a long-standing question on the signals driving the production of this critical cytokine in CD4 T cells during *in vivo* persistent infection. Given the importance of IL-21 in maintaining virus-specific CD8 T-cell responses in chronic settings (11–13) and the direct roles of IL-6 and IL-27 signaling in the regulation of virus-specific CD4 T-cell numbers and function (14, 15), our data place gp130 signaling in general and IL-6 and IL-27 signaling in particular as a key nexus of protective immunity in chronic infection.

IL-6, IL-27, and IL-21 can potently induce phosphorylation of STAT1 and STAT3 signaling (15), and STAT3 is known to be critical for IL-21 production in a range of settings (40). IL-6 and IL-27 are therefore likely to mediate much of their effect through STAT3 signaling, and indeed STAT3 expression, in T cells is essential for antibody-mediated immunity following LCMV infection (50). STAT3 is not, however, on its own essential for the accumulation of virus-specific CD8 or CD4 T cells, suggesting that STAT1 or other signaling molecules downstream of the gp130 co-receptor are also involved. Transcriptionally, it seems likely that IRF4, Aiolos, and/or Foxo3 play a role in promoting *Il21*, while counter-regulation of Aiolos by Eos may act to limit this cytokine, especially given the co-operative roles of STAT3 and Ikaros Zinc Finger transcription factors in regulating CD4 T cells (38).

One aspect not explored in the current study is precisely how IL-6 and IL-27 production are regulated during chronic viral infection. We have previously shown that IL-6 is produced in a biphasic manner during LCMV Cl13 infection with enhanced signaling once the chronic infection has been established (14). At a late stage of the infection, IL-6 appears to be primarily produced by irradiation-resistant, non-hematopoietic cells, with IL-6 expression being particularly high in follicular dendritic cells (14), stromal cells that form a network within GCs. More recently, B cells have been identified as the primary source of antiviral IL-27 during chronic infection (51). Precisely why some individuals are able to mount a neutralizing antiviral immune response to such chronic infections, and why some viruses are more effective at causing persistent lifelong infection than others, is unknown. Together, the aforementioned findings, however, suggest that cells in the B-cell follicles of secondary lymphoid tissues (e.g., follicular dendritic cells and GC B cells) are important contributors of IL-6 and IL-27, and as such, these compartments are vital anatomical sites for shaping multiple aspects of antiviral immunity during chronic infections. It may also explain why specifically targeting and disrupting B-cell follicles is such an effective strategy for enabling viral persistence, as exemplified by HIV, which preferentially infects Tfh cells (52). Further understanding the signaling required to promote immunity capable of controlling persistent infection may be vital in combating these viral strategies and/or boosting suboptimal antibody and T-cell responses in persistently infected individuals.

## Data availability statement

The datasets presented in this study can be found in online repositories. The names of the repository/repositories and accession number(s) can be found below: GSE235035 https://www.ncbi.nlm.nih.gov/geo/query/acc.cgi?acc=GSE235035 (GEO).

## Ethics statement

The animal study was reviewed and approved by Institutional animal care and use committee, University of California San Diego.

## Author contributions

JH and EZ conceptualized and designed the study. JH, BB, PB, and AD carried out experimental work. JH, BB, PB, AD and TG analyzed the data. JH and EZ interpreted the data. EZ supervised the study. JH and EZ wrote the manuscript. JH, TG and EZ edited and finalized the manuscript. All authors contributed to the article and approved the submitted version
