# Supplementary material for: IL-6 and IL-27 play both distinct and redundant roles in regulating CD4 T-cell responses during chronic viral infection
Source: Front Immunol. 2023 Jul 31;14:1221562. doi: 10.3389/fimmu.2023.1221562 (PMC10424726; doi:10.3389/fimmu.2023.1221562)
Supplement: Supplementary file 1 [file DataSheet_1.pdf]

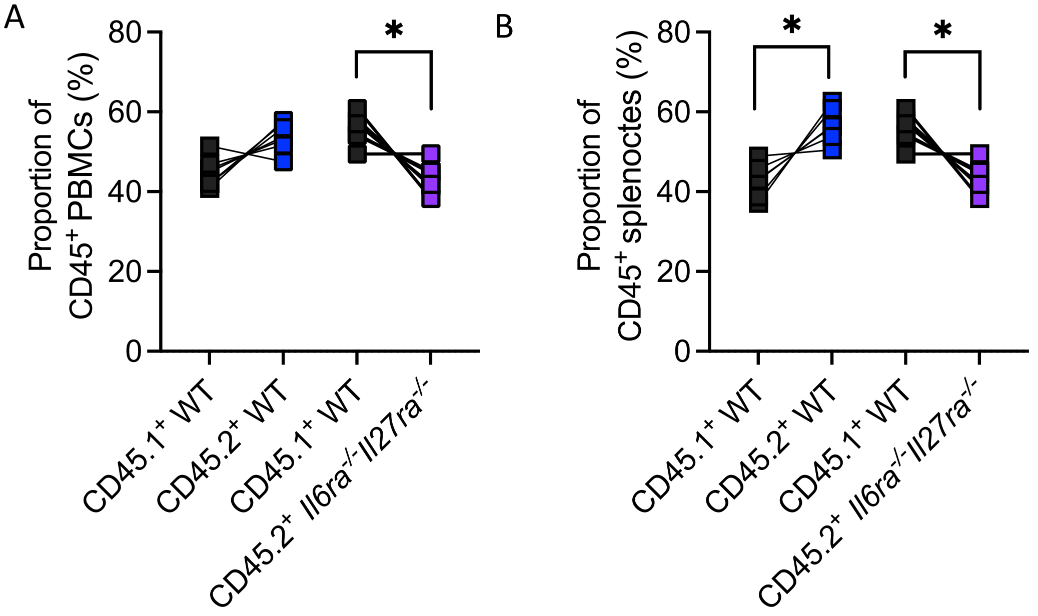


**Supplementary figure 1. Reconstitution of wildtype and dKO haemopoietic cells in mixed bone marrow chimeras.** 50:50 CD45.1$^{+}$WT:CD45.2$^{+}$ WT or *Il6ra*$^{-/-}$*Il27ra*$^{-/-}$ mixed bone marrow chimeras were generated (schematic shown in Fig. 4A). (A) Peripheral blood was taken 8 weeks after reconstitution and the frequency of CD45.1+ and CD45.2+ PBMCs determined. (B) At day 30 post LCMV Cl13 infection the frequency of CD45.1+ and CD45.2+ splenocytes was determined. Data representative of n > 4 mice per group and 3 independent repeats. Wicoxon matched pair signed rank test was used * p<0.05.
